# A hierarchy of effective teaching and learning to acquire competence in evidenced-based medicine

**DOI:** 10.1186/1472-6920-6-59

**Published:** 2006-12-15

**Authors:** Khalid S Khan, Arri Coomarasamy

**Affiliations:** 1 Division of Reproductive and Child Health, University of Birmingham, and Birmingham Women's Hospital, UK; 2 Professor of Obstetrics-Gynaecology and Clinical Epidemiology, University of Birmingham, Birmingham Women's Hospital, B15 2TG, UK

## Abstract

**Background:**

A variety of methods exists for teaching and learning evidence-based medicine (EBM). However, there is much debate about the effectiveness of various EBM teaching and learning activities, resulting in a lack of consensus as to what methods constitute the best educational practice. There is a need for a clear hierarchy of educational activities to effectively impart and acquire competence in EBM skills. This paper develops such a hierarchy based on current empirical and theoretical evidence.

**Discussion:**

EBM requires that health care decisions be based on the best available valid and relevant evidence. To achieve this, teachers delivering EBM curricula need to inculcate amongst learners the skills to gain, assess, apply, integrate and communicate new knowledge in clinical decision-making. Empirical and theoretical evidence suggests that there is a hierarchy of teaching and learning activities in terms of their  educational effectiveness: Level 1, interactive and clinically integrated activities; Level 2(a), interactive but classroom based activities; Level 2(b), didactic but clinically integrated activities; and Level 3, didactic, classroom or standalone teaching.

**Summary:**

All health care professionals need to understand and implement the principles of EBM to improve care of their patients. Interactive and clinically integrated teaching and learning activities provide the basis for the best educational practice in this field.

## Background

Evidence-based medicine (EBM) is often taught and learnt through attendance at courses, conferences, workshops, journal clubs, educational meetings, surveillance of medical literature and guidelines, and investments in textbooks [1]. Many of these activities fall under broader concepts such as Continuing Professional development (CPD) [2], Knowledge Translation (KT) [3] and Continuing Medical Education (CME). The process of EBM [4] includes formulating structured queries from specific clinical problems, searching and acquiring relevant literature, appraising it for quality and, if appropriate, applying the findings taking into account patient's own preferences and values. EBM aims to incorporate more holistic perspectives, enlisting effective implementation strategies using the influence of CME [5]. The trigger for enlisting in EBM related educational activities usually isn't the identification of an information need to resolve a particular clinical problem from a specific patient encounter, but rather a general wish to update oneself.

The different approaches to teaching and learning EBM are likely to be associated with varying levels of effectiveness in improving outcomes such as knowledge, skills, attitudes and clinician's behaviour. How can we develop a hierarchy of effective teaching and learning in imparting  and acquiring competence in EBM? We considered the evidence on  interventions for changing clinician behaviour, educational effectiveness of CME (CPD), and effective learning of EBM to produce a  meaningful rank order of practical teaching and learning activities that  are likely to be successful. Where there is absence of empirical evidence on the effectiveness of different strategies, we have relied on  theoretical considerations or existing consensus to arrive at our  conclusions.

### Literature sources

We sought for literature on the following structured question:

#### Population

Healthcare professionals.

#### Interventions and comparisons

Various methods of teaching and learning EBM and improving professional behaviour and performance.

#### Outcomes

Knowledge, skills, professional attitudes and behaviours, and health outcomes.

#### Design

Randomised controlled trials (RCT), non-randomised controlled studies, before-and-after studies, theoretical or consensus articles.

We searched the Cochrane Library, Cochrane Effective Practice and Organisation of Care (EPOC) Group database [6], MEDLINE, EMBASE, ERIC, and Best Evidence Medical Education (BEME) database to identify relevant articles using "evidence based medicine", "continuing medical education", "continuing professional development" and their word variants as search terms. We consulted with experts directly as well as via an Internet discussion forum [7] and sifted through our personal files on EBM and medical education. The searches were updated in 2006.

Searching for papers on empirical evidence was guided by selection criteria based on the structured question above. We reviewed existing systematic reviews of primary literature on the effectiveness of CME, e-learning and EBM teaching activities. Searching for papers concerning educational theory and principles in electronic databases was not as straightforward because of inconsistent indexing and the absence of a specific keyword for the relevant publication types. Several of these concepts have been developed in areas other than CME and EBM. Conducting a broad literature search to capture every single potentially relevant paper would have been impossible if not inappropriate, especially since precise estimation is not the objective of such a review. Once a set of relevant concepts had been elucidated, there was no additional value in reviewing more papers explaining the same concept. This is known as theoretical saturation [8], a principle that guided our search and selection for papers on theories and principles. We discuss and summarise our finding and deliberations below. After considering empirical evidence, educational theory and related educational principles, we develop a hierarchy for teaching and learning of EBM.

## Discussion

### Empirical evidence

New teaching and learning activities and methods can be evaluated against a contemporaneous control educational strategy, or baseline before the new educational activity, using measures of patients' health gains (clinical outcome) or participants' learning achievements (educational outcome) as endpoints for evaluation of effectiveness and impact [9-11] (Table 1). Learning achievement can be assessed separately for knowledge, skills, attitudes, and behaviour [10]. Knowledge relates to issues such as remembering materials as well as grasping the meaning, for example, defining and understanding the meaning of Numbers Needed to Treat (NNT). If this knowledge can then be applied accurately to given problems, then this will be regarded as a gain in critical appraisal skills; for example, the ability to generate NNTs when baseline risks and relative risks are provided. Spontaneously acknowledging a need for the use of a certain piece of knowledge or skill in practice will be regarded as a change in attitude, for example, recognising without prompting the need for different NNTs for different clinical scenarios and intending to calculate the respective NNTs for different risk levels. Finally, a change in behaviour occurs when one seeks the necessary information and applies the knowledge and skills to solve the issue in practice, for example, searching the literature, finding relevant baseline risks and relative risks and calculating necessary NNTs to guide clinical practice. Ultimately by consistently applying these findings in practice, patients' outcomes can be expected to improve. Insistence that these competencies must translate into improved clinical outcomes or outputs for our patients, or that the proof can only be considered valid if generated through RCT is not the subject of our commentary. The empirical evidence is summarised below looking at various elements of interactive and integrated teaching and learning. Some readers may be concerned that strength of the empirical evidence may be overstated, and if they need assistance in interpreting the results of systematic reviews they may wish to consult existing texts on appraising such reviews [12-14].

#### Interactive vs didactic education activities

EBM teaching and learning during post-graduate and continuing medical education takes place in the CME context. A traditional CME event can be didactic, interactive or mixed and can be either a single or sequenced event [15]. Didactic sessions comprise of lectures, but may include question and answer periods. The interactive sessions are those that involve some form of interaction amongst the participants, which may take the format of small-group work, role-play, case discussions, or the opportunity to practice skills [16]. Mixed sessions include both didactic and interactive elements. A Cochrane review [16], which updated previous systematic reviews [15,17], evaluated educational activities such as lectures, workshops and courses. There were 32 studies (35 comparisons) that evaluated educational meetings of various types against no intervention, and of these, 24 studies (26 comparisons) reported significant improvement in professional practice (in at least one major outcome measure). Eight studies reported on patient health outcomes, and three of these found a significant improvement with educational meetings compared to no intervention. Overall, the evidence suggested that educational interventions can improve both professional practice and health outcomes. However, there was substantial heterogeneity in the types of educational interventions and their effects, necessitating an analysis beyond simply focussing on the overall results, and raising the question: What particular types of educational activities produce the benefit noted in some of the studies in the systematic reviews, and what features are associated with no or limited improvements in outcomes?

Eight studies, consisting of seven RCTs and one non-randomised study, evaluated interactive workshops compared to either no intervention (six studies) or other formats [16], In 7/8 studies, interactive workshops were found to result in significant improvements in practice in at least one major outcome measure. One study reported patient outcome and found that interactive sessions resulted in a significant reduction in asthma symptoms among paediatric patients. On the other hand seven RCTs evaluated a presentation or a lecture targeted at specific behaviours [16], In six of seven studies, there were no significant differences between the trial arms. One study reported a statistically significant, but small, effect in one of four skin cancer screening behaviours. A direct comparison between interactive workshops and didactic presentations reported no differences between the two groups. Although the available evidence did not allow an examination to assess what makes interactive workshops more effective than others, the evidence is clear: interactive workshops can improve education and patient outcomes whilst didactic teaching alone is unlikely to result in improvements. Therefore educational activities with an interactive format should rank high in any hierarchy of effective EBM teaching.

#### Educational activities based on e-Learning

E-learning technologies are increasingly being used to develop interactive curricula on key aspects of EBM [18,19]. The Internet provides an important forum for EBM teaching and learning and its role is likely to expand in the future. It provides an efficient and increasingly interactive delivery system that can handle complex and layered information. Furthermore, it is not limited by time or geography and can be integrated into practice with easy availability of information and communication technology within clinical practice areas. Moreover, the learner sets the pace and the depth of learning. Self-assessment and feedback, as well as interactivity and networking with other participants, are all possible with eLearning. What is the evidence for the effects of eLearning? We identified a systematic review that had identified 16 RCTs that evaluated the effectiveness of Internet-based education in medical students or practicing healthcare professionals [20]. Six studies showed a positive change in participants' knowledge, and three showed a change in practice in comparison to traditional formats. There were no data on health outcomes. These results show that e-Learning can be effective – however, as the evidence relates to a rapidly changing technology and there is extensive heterogeneity in teaching methods, delivery systems, assessment methods [21,22] as well as other features of the existing studies, it is not possible to establish which elements contribute to an effective e-Learning strategy in EBM.

#### Integrated vs stand alone educational activities

We carried out a systematic review [23] of existing literature on the effectiveness of teaching EBM to post-graduates, to evaluate if the incorporation of teaching into clinical practice had any impact on outcomes. The review [23] included randomised, non-randomised controlled as well as before-and-after comparison studies, although greater weight was given to randomised evidence in the inferences. There were 23 studies, of which three were randomised trials, seven were non-randomised controlled studies and 13 were before-and-after comparison studies. We compared classroom – either didactic, interactive or mixed – versus clinically integrated teaching. Eighteen studies (including two randomised trials) evaluated a standalone teaching method, whilst five studies (including one randomised trial) evaluated a clinically-integrated teaching method. Due to poverty of reporting and substantial heterogeneity in populations, teaching methods, outcome definitions, assessment tools (most unvalidated), and methodological quality, we carried out a qualitative data synthesis in the form of what is often described as 'vote-counting'. Synthesis was conducted within broad subgroups of teaching methods and educational outcomes stratified by study methodology. This type of approach to minimise bias in 'vote-counting' by incorporating quality has previously been used to synthesise heterogeneous results [24]. Standalone teaching improved knowledge, but not skills, attitudes or behaviour. Clinically-integrated teaching, on the other hand, improved knowledge, skills, attitudes and behaviour. None of the studies evaluated patient outcomes. EBM teaching integrated into clinical practice was, therefore, found to be superior to classroom teaching in improving educational outcomes, including positive changes in attitudes and behaviours of clinicians.

### Theoretical considerations

**Figure 1:**
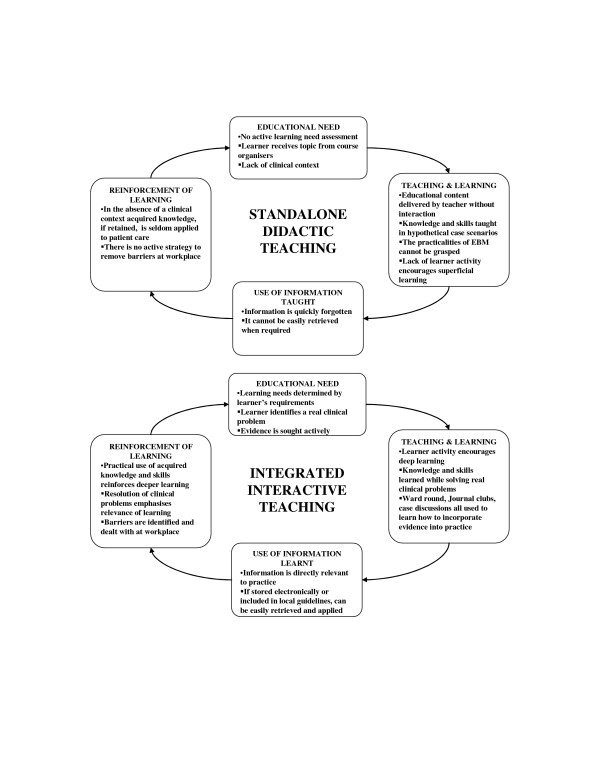
Reasons why clinically-integrated-interactive teaching may achieve better outcomes in comparison to standalone-didactic teaching.

What is the best way to learn EBM? Can learning of EBM improve not just basic educational outcomes such as knowledge and appraisal skills, but also substantial outcomes such as attitudes and clinician's behaviour? Can EBM learning result in improved patient outcomes? There are several theoretical reasons why clinically-integrated interactive teaching of EBM may produce better results in comparison to standalone didactic teaching [25]. These are explored in Figure 1. If learners are fully immersed in educational experience, feel relaxed alertness thereby eliminating fear while maintaining challenge, and actively participate in processing information, they will consolidate and internalise the learnt materials effectively [26-28]. Changes in their attitudes are likely to be important in bringing about sustained changes in behaviour, which is what will ultimately benefit patient care.

In a rapidly changing world, health care practitioners need to approach their profession with a view to lifelong learning [29]. They need to identify their educational needs and develop a strategy to meet those needs in realistic and effective manner. This is likely to be achieved best with an *a priori *outline of a personal learning plan, which can serve as a vehicle for guiding educational activities over a specified time period when the progress can be assessed and a follow up learning plan formulated. In generating a learning plan health care practitioners should identify their own needs and set learning objectives accordingly. Their personal learning plan is based on the principles of adult learning [30-33], whereby they take responsibility for their own learning through a systematic programme of acquisition, renewal, upgrading and completion of knowledge and skills outlined in their professional development objectives. The objectives should be based on what they and their organisation need to learn. If there is cognateness between individual and organisational learning needs, the learning plan is more likely to be supported [34]. There should be a practical, flexible and achievable strategy for learning which has a realistic hope of meeting the learning objectives within the limits of the resources available to the individual and their organisation. The choices should be driven by elements of effective CME outlined above.

Ideally, the plan for learning should be entirely voluntary and it should be driven by personal motivation of learner [35]. Individuals should make the choice of the subject matter they wish to learn, the manner in which they will go about learning it, and they should learn independently without the need for close supervision. The ability to fulfil learning objectives will depend on critical reflection on experiences throughout the planned learning period [35]. Therefore the plan should delineate learning outputs and outcomes to assess whether learning objectives have been met. The experience should help them critically analyse their performance against standard criteria using both formative and summative style assessments [36]. In this manner a self-directed individual can be nurtured in the course of a CME cycle. Such an approach when applied diligently can help with personal development, revalidation etc., but above all it has a real chance of making a difference to our patients' outcomes.

Below we list elements of CME that we consider would enhance the value for the learner, and ultimately for their patients. Although some of the elements are based on empirical evidence, the others are based on educational theory and practice.

 Learning using an interactive approach [16].

 Learning incorporated into clinical practice [23].

 Sequenced events that aid reinforcement, rather than single or episodic CME events.

 Courses that identify and take into account learner needs.

 Courses that take place in the context of CPD [2] and KT [3], as well as local and national service objectives.

 Multi-faceted strategies in teaching and learning [37,38].

 Course that give individual feedback and the opportunity for self-assessment.

Various other effective approaches exist, such as educational outreach for prescribing, reminders, opinion leaders, as well as audit and feedback [37,38]. Moreover, not surprisingly, multifaceted interventions targeting different barriers have been shown to be more likely to be effective than single interventions [37,38]. These, when combined with effective teaching and learning, are likely to bring about desired outcomes for EBM.

Practitioners should view CME concerning teaching and learning of EBM in a holistic manner. CPD encompasses traditional CME as well the acquisition of other skills such as administration, management, teaching, and communication, and embodies elements such as self-directed, patient-centred and individualised learning as well as continuing appraisal [2]. Work based learning and KT are elements of in-house CPD that focus on knowledge and skill application in the workplace [39]. KT is the process whereby information is transferred to clinicians and applied in practice, a process that requires understanding of complex interactions between various individuals (medical and non-medical) and organizations [3]. It focuses on health outcomes and changing behaviour [3]. EBM, which can change physician's practice [23] and thus holds the potential to change health outcomes, can be a critical tool in KT. Whilst CME or CPD is primarily located in teaching settings, KT activities are centred in practice settings, and place a greater focus on group or team learning [3]. KT is an element of CPD that is receiving wider attention in the field of CME.

### A hierarchy of effective EBM teaching and learning

Based on educational evidence, theory and principles we propose a hierarchy of teaching and learning methods for EBM (Table 2).

#### Level 1: Interactive and clinically integrated teaching and learning activities

Substantial empirical evidence exits to support interactive teaching over didactic teaching. Clinically integrated EBM teaching is more effective than classroom teaching because it interrelates and unifies clinical subjects with clinical epidemiology creating a meaningful whole. Taking into account that educational theory and evidence on changing physician's behaviour are consistent with clinically integrated teaching being better than classroom teaching and learning. Therefore, an interactive and integrated learning should be the ideal that EBM practitioners should aim for as this probably represents the most effective way of learning. It is reflective of practice, it allows identification of gaps between current desired levels of competence, it identifies solutions that are practically testable, and it allows re-evaluation with the opportunity for further reflection and continuum of learning [39]. Interactivity encourages deeper learning, which is important for understanding, manipulation and transference of learnt materials into practice.

#### Level 2: (a) Interactive, classroom based teaching, or (b) didactic, but clinically integrated teaching

Many of the modern teaching activities fall into the former category in which although teaching is located in a classroom, efforts are made to make the sessions interactive with small-group work, role plays and case discussions. Activity is the key to effective training, and this is the defining feature of interactive learning. On the other hand, teaching can be didactic but clinically integrated – an example of this would be a one-way discourse by a clinical teacher to students on a ward-round (classical bed-side teaching). Basing the discourse on a patient problem is likely to help with showing the relevance and application of the EBM knowledge. Such teaching can be easily turned into the interactive format with the likelihood of greater educational benefit. Such an approach, with live video linking and ability to interact with large groups at remote sites, makes it possible to convert classical teaching methods for wider application. E-health is an emerging field and teaching and learning medicine via videoteleconferencing will no doubt develop in the future [40,41].

#### Level 3: Didactic, and classroom or standalone teaching

Many traditional teaching activities fall into this category, and they are unlikely to be effective in improving clinicians' performance or heath outcomes for the patients. Whilst they may have their own benefits (such as allowing networking between those with an interest in a particular topic), unless at least they contain elements of interactivity (for example, small group work or case discussions), their worth is likely to be limited. This is because lack of interactivity encourages superficial (rote and regurgitation) learning.

### Limitations and barriers

The concepts behind the above hierarchy are gaining in popularity. However, it needs to be recognised that many caveats need consideration. We believe that we have taken into account both empirical evidence and theory judiciously in generating this ranking of teaching and learning activities. However, there may be concern that empirical evidence has been weighted more than theoretical considerations. On balance, we think that the weight to be attached to theoretical evidence should be considered very carefully, as theory so often does not materialise as expected when put into practice. Thus, we are confident that our emphasis on empirical evidence, particularly on evidence from a systematic synthesis of literature, is justified.

Decision-making about choice of teaching and learning methods should be guided by comparison of effects and costs associated with various educational strategies. Our aim was indeed to generate numerical measures of the effect size(s), however, due to poverty of reporting and substantial heterogeneity (in populations, teaching methods, outcome definitions, assessment tools and study quality, amongst other features), we were unable to provide any statistical summaries. Two very important issues where there were no or scant data relate to long term effects of the teaching methods and the effect on clinical outcomes. Pragmatic decisions have to be made based on the information available and in the absence of meta-analytic summaries, qualitative syntheses within broad subgroups of teaching methods and educational outcomes stratified by study methodology minimise biased inferences [24]. When dominance can be so obviously demonstrated, a basic economic evaluation does not require statistically sophisticated analyses. For example, in a recent study [42], the average cost of providing a critical appraisal workshop was approximately £250 per person but no improvement was demonstrated in knowledge or attitudes. These findings challenge the policy of funding 'one-off' educational interventions aimed at enhancing EBM. Cost-effectiveness analyses are often simple and straightforward when the issue of dominance is not finely balanced [43]. Is it reasonable to propose that standalone teaching should be abandoned, when an effort without desirable benefits does incur substantial costs? We think yes.

There are many barriers to the feasibility of an interactive and integrated approach and to its acceptability to both teachers and learners. Those who endeavour to embark on higher level of EBM teaching methods in our hierarchy will need to study these carefully as part of an implementation plan [44-47]. These methods may be considered similar to an innovation in many settings with the need for various phases for embedding them in practice [48], For example within problem- based learning, there has been discussion about the utility of introducing of interactive approaches to learners who have yet to acquire a baseline level of knowledge. Interactive approaches are also seen as challenging across different cultures that have a tradition of didactic teaching. In these situations, methods lower down in our hierarchy may provide the pre-requisite knowledge or encouragement to learners before they engage in interactive and clinically-integrated teaching. It is important to remember that education concerning EBM can take one through the key initial stages of change before one is prepared to adopt EBM in practice. EBM is a strategy for just-in-time learning [49] that increasingly is possible in a clinical environment. Teachers and learners should carefully examine their learning environment and circumstances when developing an implementation strategy [50] for their chosen EBM related educational activities.

## Summary

As the stated aim of EBM is to benefit patient care, it becomes necessary that teachers and learners of EBM consciously find ways of integrating and incorporating teaching and learning into routine clinical practice [51]. Where resources and facilities are available, such learning can form part of a real-time ward-round or clinic with the dual purposes of learning EBM skills and attempting to improve patient care with best available evidence [44,52]. If the provisions for a real-time teaching are not available, then even traditional learning settings, such as a journal club [39,46,52-54], can be adapted to be based on real and current clinical problems, thus illustrating the process is not a mere academic exercise, but it informs patient care [52]. These teaching and learning methods have the potential to demonstrate how that those receiving care could make decisions (wherever possible), informed by the knowledge of their care providers, within the context of available resources. Learning of EBM should therefore be moved from classrooms to clinical settings. Indeed this approach should be generalisable to other clinical topics – not just EBM – and integration of teaching and learning into practice should be considered for all topics in health care too.

## Competing interests

The authors have received honoraria for teaching EBM from health technology (including pharmaceutical) manufacturers.

## Authors' contributions

KSK and AC contributed to the concept and wrote and revised the manuscript.

## Pre-publication history

The pre-publication history for this paper can be accessed here:



## Figures and Tables

**Table 1:** Outcomes and impact of evidence-based medicine (EBM) Teaching and learning

Kirkpatrick scale*	**Outcome measures**
Participation	*Learners' views on the learning experience during educational activity (e.g. post teaching questionnaire)*
Modification of attitudes/perceptions	*Change in attitudes or perceptions between participant groups as a result of educational activity (e.g. difference between pre and post teaching questionnaire on attitudes)*
Modification of knowledge or skills	*Change in knowledge or skills between participant groups as a result of educational activity (e.g. difference in performance between pre and post tests)*
Behavioural change	*Transfer of learning to the workplace or willingness of learners to apply new knowledge or skills (e.g. difference between pre and post questionnaire on attitudes)*
Change in organisational practice	*Wider changes in the organisation/delivery of care, attributable to educational activity (e.g. updating of guidelines or care pathways)*
Benefit to patients/clients	*Improvement in the health and wellbeing of patients as a result of educational activity (e.g. audit of outcomes in practice)*

* adapted from Belfield et al [9]

**Table 2:** A hierarchy of evidence-based medicine (EBM) teaching and learning

Level 1:
Interactive, and clinically integrated teaching and learning activities

Level 2:
(a) Interactive, classroom based teaching and learning activities
(b) Didactic, but clinically integrated teaching and learning activities

Level 3:
Didactic, and classroom or standalone teaching and learning activities
